# Predictive value of m5C regulatory gene expression in pancreatic adenocarcinoma

**DOI:** 10.1038/s41598-021-96470-w

**Published:** 2021-09-01

**Authors:** Xiao Yu, Qiyao Zhang, Fang Gao, Menggang Zhang, Qingyuan Zheng, Yuting He, Wenzhi Guo

**Affiliations:** 1 Department of Hepatobiliary and Pancreatic Surgery, The First Affiliated Hospital of Zhengzhou University, Zhengzhou, 450052 Henan People’s Republic of China; 2 Key Laboratory of Hepatobiliary and Pancreatic Surgery and Digestive Organ Transplantation of Henan Province, The First Affiliated Hospital of Zhengzhou University, Zhengzhou, 450052 Henan People’s Republic of China; 3 Open and Key Laboratory of Hepatobiliary & Pancreatic Surgery and Digestive Organ Transplantation at Henan Universities, Zhengzhou, 450052 People’s Republic of China; 4 Henan Key Laboratory of Digestive Organ Transplantation, Zhengzhou, 450052 People’s Republic of China; 5 Health Management Center, Binzhou People’s Hospital, Binzhou, 256600 People’s Republic of China

**Keywords:** Cancer, Computational biology and bioinformatics, Oncology

## Abstract

Pancreatic adenocarcinoma (PAAD) is the most malignant digestive tumor. The global incidence of pancreatic cancer has been rapidly trending upwards, necessitating an exploration of potential prognostic biomarkers and mechanisms of disease development. One of the most prevalent RNA modifications is 5-methylcytosine (m5C); however, its contribution to PAAD remains unclear. Data from The Cancer Genome Atlas (TCGA) database, including genes, copy number variations (CNVs), and simple nucleotide variations (SNVs), were obtained in the present study to identify gene signatures and prognostic values for m5C regulators in PAAD. Regulatory gene m5C changes were significantly correlated with *TP53, BRCA1, CDKN2A,* and *ATM* genes, which play important roles in PAAD pathogenesis. In particular, there was a significant relationship between m5C regulatory gene CNVs, especially in genes encoding epigenetic “writers”. According to m5C-regulated gene expression in clinically graded cases, one m5C-regulated genes, *DNMT3A*, showed both a strong effect on CNVs and a significant correlation between expression level and clinical grade (*P* < 0.05). Furthermore, low *DNMT3A* expression was not only associated with poor PAAD patient prognosis but also with the ribosomal processing. The relationship between low *DNMT3A* expression and poor prognosis was confirmed in an International Cancer Genome Consortium (ICGC) validation dataset.

## Introduction

Pancreatic adenocarcinoma (PAAD) is the most malignant tumor of the human digestive system. New cases of PAAD in the United States rank 10th among men, 9th among women, and 4th in cancer mortality according to data released by the American Cancer Society in 2019^1^. For malignant tumors in China, PAAD ranks eighth for urban men and fifth for the mortality rate in large cities according to data from the National Cancer Center of China^2^, with a 5-year survival rate of less than 5%^3,4^. Globally, PAAD incidence and mortality increases with age and is slightly higher among men, although mortality has increased in both genders over the past few decades^3,5–8^. Despite the identification of some risk factors such as smoking, genetic diabetes, alcohol consumption, and lack of exercise, the causes of PAAD remain poorly understood^9–14^. Exploring potential prognostic biomarkers and mechanisms that drive pancreatic cancer are therefore urgently required.

RNA modifications play a pivotal role across many biological processes^15–17^. Among the 150 types of RNA modifications discovered, methylation has been identified as a common phenomenon and a key regulator of gene transcript expression, with 5-methylcytosine (m5C) being one of the most prevalent^18,19^. With the application of high-throughput sequencing techniques for detecting m5C RNA modifications (e.g., bisulfite sequencing), a whole-transcriptome map of m5C sites has become available in which m5C mainly appears in the anticodon loop and variable region of tRNAs and rRNAs and the coding sequences of mRNAs^20–24^. m5C deposition occurs through a methyltransferase complex involving three homologous factors, including methyltransferases (“writers”), demethylases (“erasers”), and m5C binding proteins (“readers”). m5C affects RNA structural stability and translational efficiency like other nucleobase modifications, and research has additionally revealed a potential role in promoting mRNA export and regulating tissue differentiation^25,26^. Dysregulation of m5C has been implicated in multiple abnormal cellular processes and human cancers^27^. For instance, tumor malignancy has been strongly correlated with levels of m5C in the DNA from breast cancer patient tissue. Measuring m5C levels in myeloid malignancies could also act as a valuable diagnostic and prognostic tool, not only for tailoring therapies but also assessing responses to anti-cancer drug activity and toxicity^28–30^.

Little is currently known about the relationship between m5C-related genes and PAAD. Based on The Cancer Genome Atlas (TCGA) database, researchers can easily access the gene expression, copy number variations (CNVs), and simple nucleotide variations (SNVs) data of human cancers. Both CNVs and SNVs can play crucial roles in cancer progression, but how the CNVs and SNVs of m5C-related genes contribute are poorly understood^31^. In the present study, we retrospectively analyzed the gene signatures and prognostic values of m5C regulators in PAAD using patient gene expression, CNV, and SNV data from the TCGA database. Changes to m5C regulatory genes were significantly associated with tumor stage, and a strong relationship between m5C regulatory gene CNVs and pancreatic cancer patient survival was established. These findings enabled the discovery of genetic alterations to m5C regulatory genes that contribute to PAAD patient outcomes and enhance our understanding of m5C epigenetic regulation in PAAD.

## Results

### m5C regulatory gene mutations in PAAD

**Figure 1:**
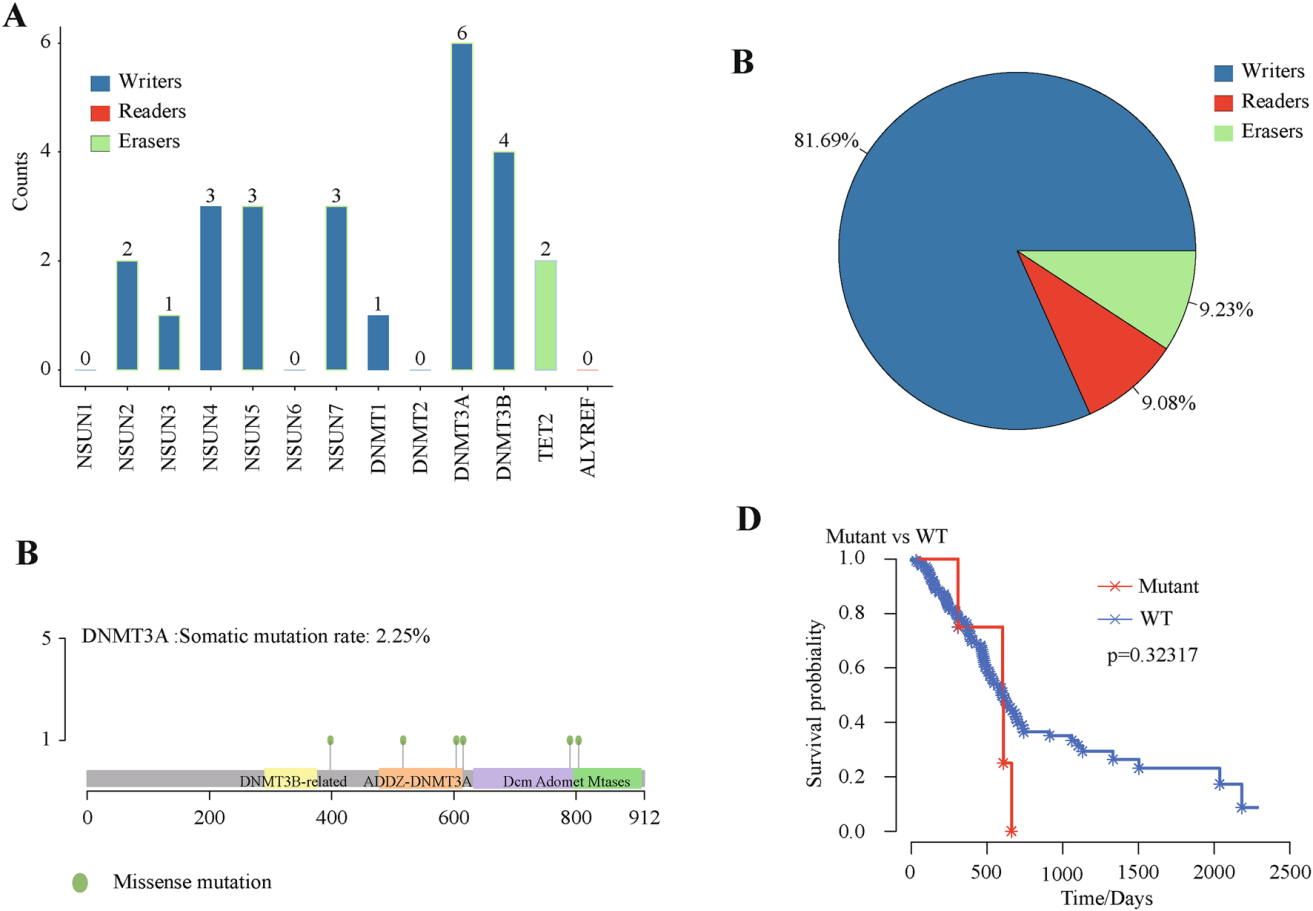
CNVs, mutations, and SNVs of m5C regulatory genes in PAAD. A total of 363 PAAD patients from TCGA were analyzed. (**A**) Mutation frequencies in m5C regulatory genes with indicated functions. (**B**) Mutation sites in *DNMT3A*. (**C**) Seven functionally altered genes and patient survival. *P* value did not reach significance due to the small number of mutations. (**D**) CNV statistics of m5C regulatory genes in PAAD samples. Writers accounted for 81.69%, which was highest. Readers and erasers made up almost the same proportion at approximately 9%.

In the sequencing data set of 363 PAAD patients, m5C regulatory gene mutations appeared in 13 independent samples (Table 1). Further, these 13 PDAC samples were used to analyze the mutation characteristics of the m5C genes. The clinical information of these samples was shown in Supplementary Table 1. Compared with the eraser and reader genes, writer gene had a greater mutation frequency, as reader gene mutations were not detected (Fig. 1A). Six of the samples revealed a higher mutation frequency in the writer gene *DNMT3A*. In 183 PAAD samples with CNV data, m5C regulatory genes showed a high frequency of CNV (Fig. 1B), although CNVs within *NSUN1* and *DNMT2* genes were undetectable. For example, the reader gene *ALYREF* had a CNV event frequency of 18.48%, followed by the writer gene *NSUN4* with a frequency of 15.22%. The eraser gene *TET2* had the lowest frequency at 6.42% (Supplementary Table 2). Among them, the writer gene *DNMT3A* showed the highest mutation level (Fig. 1C).

**Table 1:** Relationship between changes in m5C regulatory genes and high-frequency PAAD-related genes.

			Without SNV and CNV	With SNV and CNV	*X*^*2*^	*P*
*ATM*		WT	148	0	104.012609	2.0103E−24
	n = 151	Alternation	0	3		
*BRCA1*		WT	149	0	84.1791333	4.5192E−20
	n = 151	Alternation	0	2		
*CDKN2A*		WT	150	1	18.3742163	1.815E−05
	n = 151	Alternation	0	1		
*TP53*		WT	52	49	37.016707	1.1712E−09
	n = 151	Alternation	0	50		
		WT	148	0

**Figure 2:**
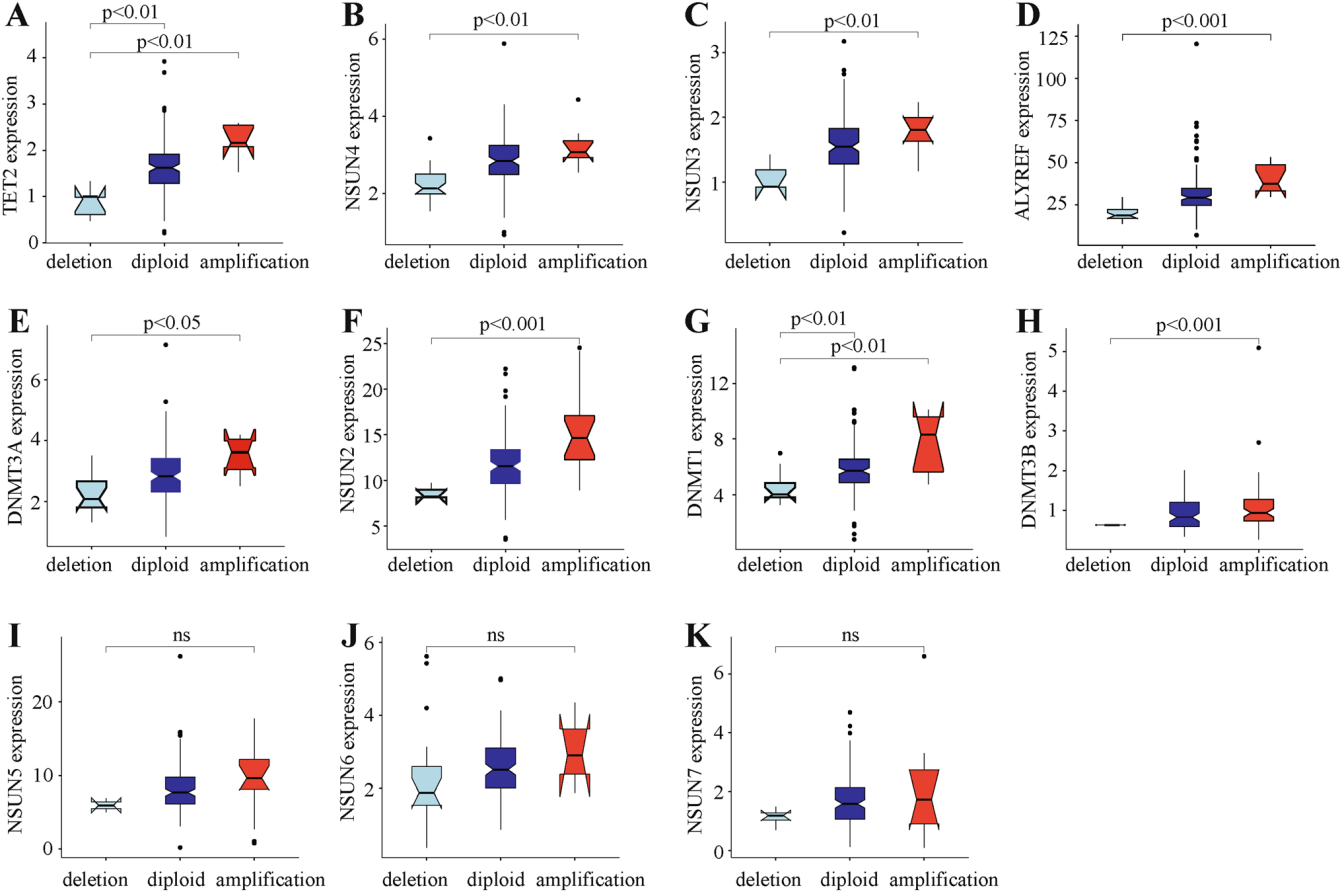
m5C regulatory gene correlation to clinicopathological molecular characteristics. (**A**–**H**) Relationship between CNVs and expression levels of m5C regulatory genes (significance *P* < 0.01). (**I**–**K**) Relationship between CNVs and expression level non-significant (ns) m5C regulatory genes.

These results showed that in 177 PAAD samples, two out of 13 m5C regulatory genes were hardly detected. Excluding these two genes, mRNA expression levels of the remaining 11 m5C regulatory genes significantly correlated with their CNV patterns. Increased copy numbers for eight genes, which were distributed across all m5C regulation categories, were associated with higher mRNA expression. Deletion mutants also resulted in decreased mRNA expression. Additionally, three out of 11 m5C regulatory genes had no significant relationship with CNV expression and were concentrated only in writers genes (Fig. 2). The expression of all eraser and reader genes were significantly related to CNVs. We next used seven functionally altered genes to predict PAAD patient survival, but only uncovered a small number of mutations without predictive statistical significance (Fig. 1D).

### m5C regulatory gene changes are related to molecular clinicopathological characteristics

Since *TP53, BRCA1, CDKN2A,* and *ATM* genes play crucial roles in the pathogenesis of PAAD, we investigated their relationship with m5C regulatory genes. Interestingly, variations in m5C regulatory genes associated significantly with alterations to *TP53, BRCA1, CDKN2A,* and *ATM* (Table 1).

### Association between m5C regulatory genes and PAAD survival

**Figure 3:**
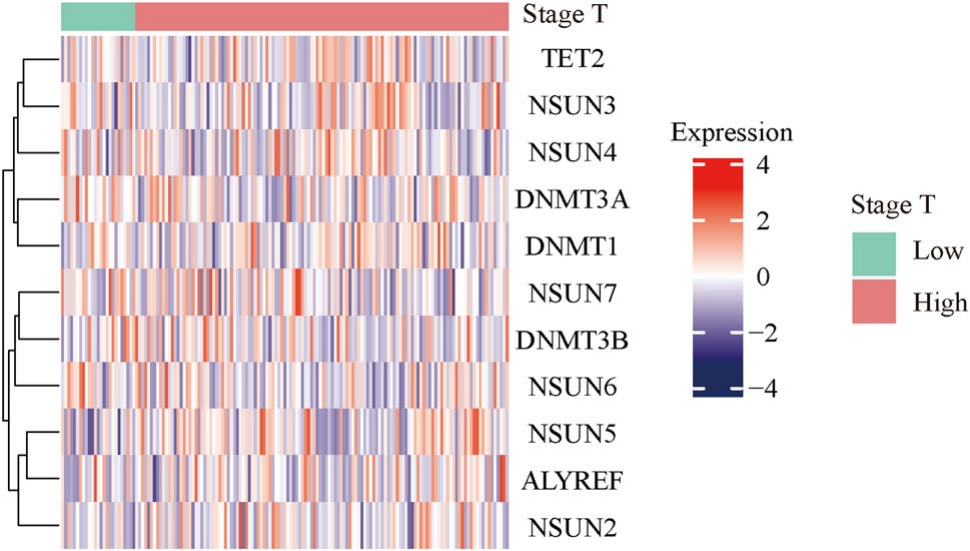
Heat map clustering between m5C regulatory gene expression and clinical tumor staging.

**Figure 4:**
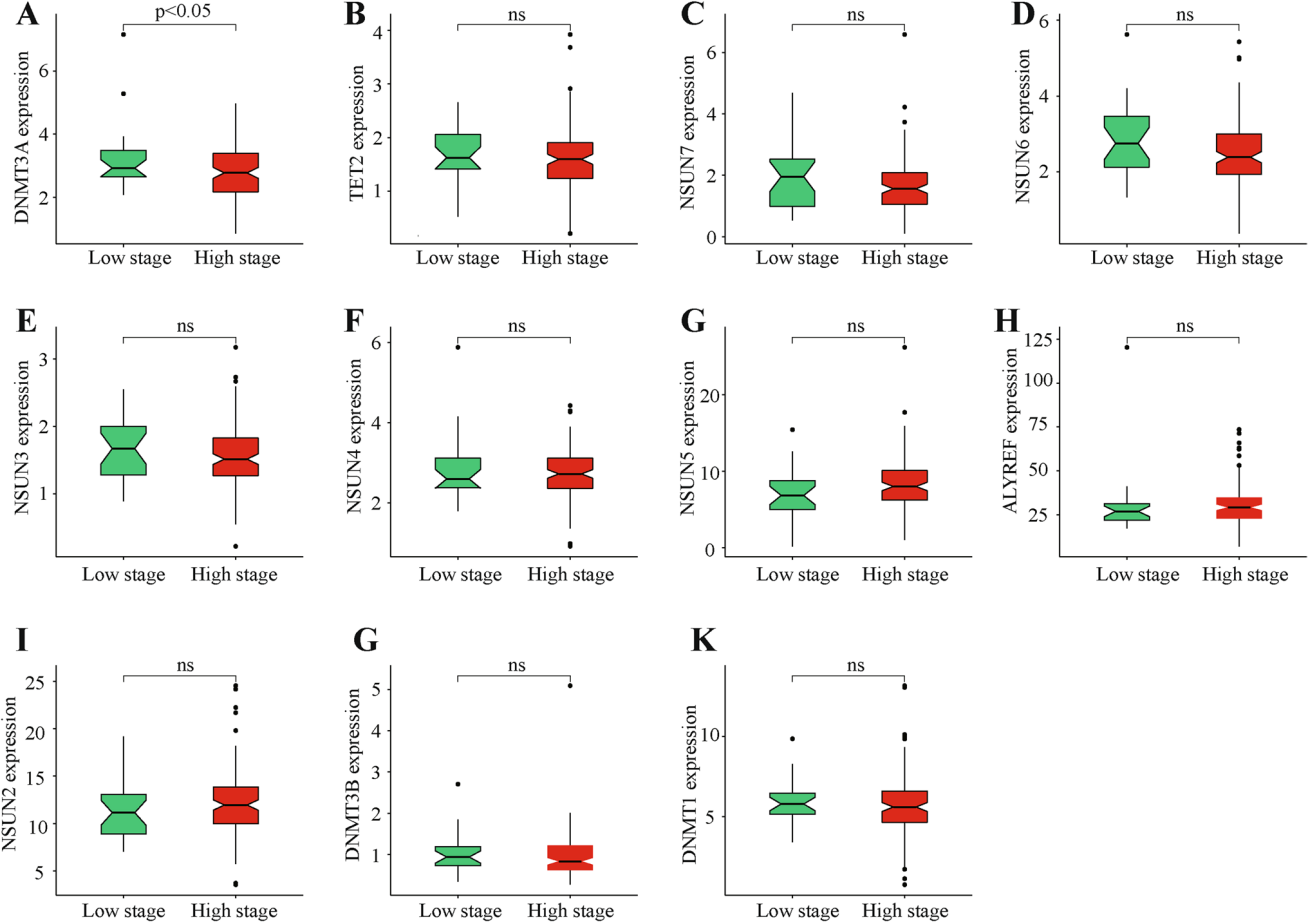
m5C regulatory gene expression in different clinically graded PAAD cases. (**A**) Comparison of m5C regulatory gene expression in different clinically graded cases. *DNMT3A* expression showed a significant negative correlation with patient clinical grade (significance *P* < 0.05). (**B**–**K**) Comparison of m5C regulatory gene expression in different clinically graded cases that were non-significant (ns).

We then analyzed and clustered m5C regulatory gene expression in different clinically graded cases (Fig. 3). These results indicated that only one out of 11 m5C regulatory genes, *DNMT3A*, significantly correlated with the clinical grade of patients in that its high expression associated with high staging (Fig. 4). As previously described, *DNMT3A* also had the highest mutation rate and a positive correlation between CNV changes and expression level.

**Figure 5:**
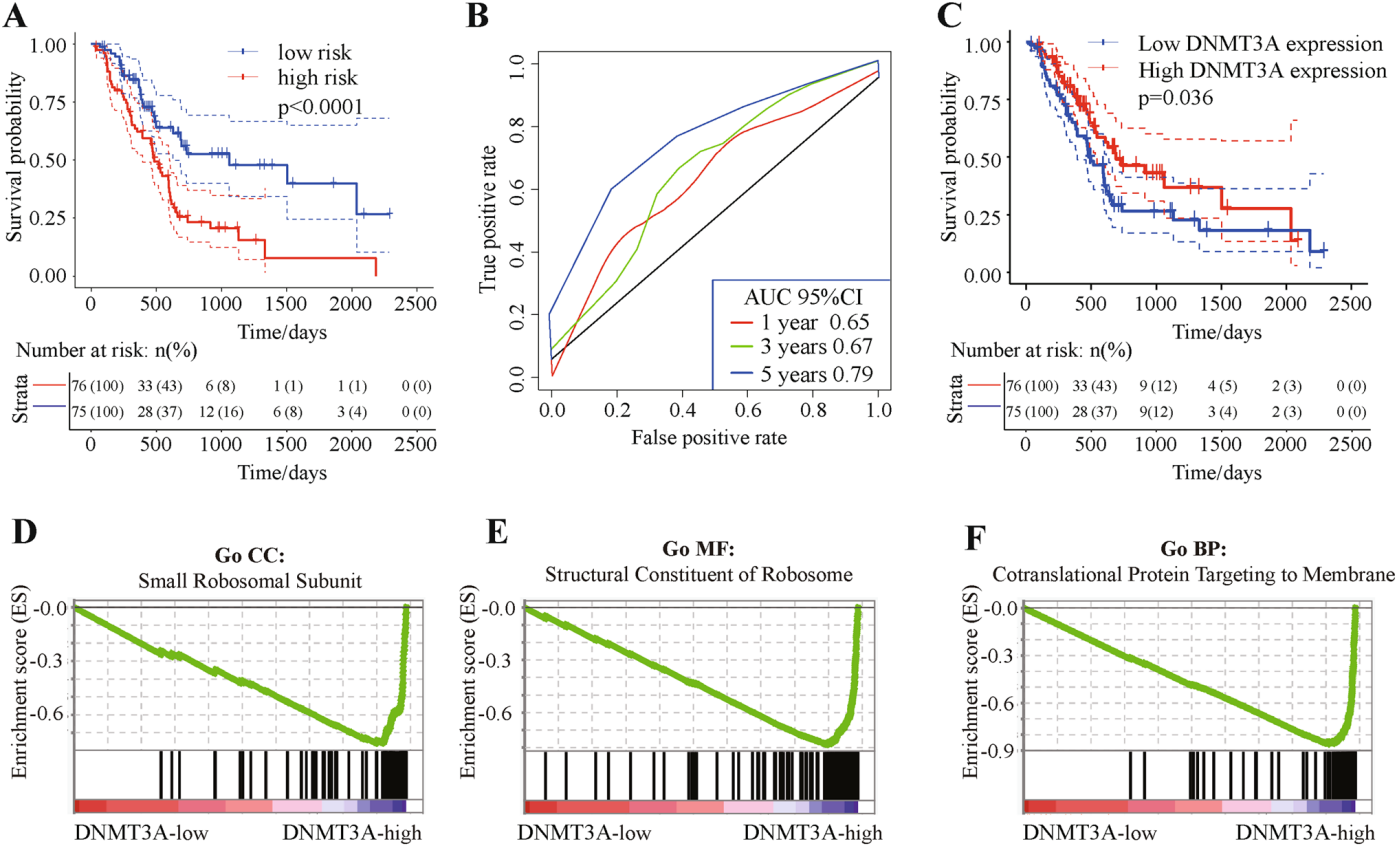
Relationship between m5C regulatory gene expression and patient prognosis, with *DNMT3A* gene functional enrichment analysis. The impact of 13 m5C regulatory genes on patient prognosis using multivariate Cox regression. (**A**,** B**) Multivariate Cox regression of survival (*P* < 0.001) and AUC curves (AUC curve areas > 0.65). (**C**) Relationship between *DNMT3A* expression and patient prognosis (*P* = 0.036). (**D**–**F**) GSEA enrichment analysis of the *DNMT3A* Gene showing low *DNMT3A* expression related to ribosomal processing.

Given the relationship between clinical classification and patient prognosis, we believed that m5C regulatory gene expression would be related to patient prognosis. However, no significant correlation between m5C regulatory gene CNVs and patient prognosis was observed. In order to correlate the expression of some m5C regulatory genes with CNVs, we used single factor Cox regression to explore the relationship between m5C regulatory gene expression and patient prognosis. The expression of two genes showed a significant positive correlation with patient prognosis (*P* < 0.05), including *DNMT3A* (Table 2). Among these two genes, the expression level of *DNMT3A* was significantly related to CNV changes (Table 2). Using multivariate Cox regression analysis, we next found that m5C regulatory gene expression can significantly predict patient risk using AUC at 1, 3, and 5 years, with all AUC values greater than 0.65 (Fig. 5A,B). These results demonstrated that the expression of m5C regulatory genes can be used as a prognostic marker for pancreatic cancer.

**Table 2:** Univariate Cox regression exploring the relationship between different m5C regulatory gene expression levels and patient prognosis.

	Beta	HR	(95% CI for HR)	Wald test	*P*-value	CNV sig
*DNMT3A*	− 0.31	0.74	(0.57–0.95)	5.6	0.018	Yes
*NSUN6*	− 0.3	0.74	(0.57–0.96)	5.2	0.022	No
*NSUN7*	− 0.21	0.81	(0.64–1)	3.2	0.071	No
*NSUN2*	0.044	1	(0.98–1.1)	2.1	0.15	Yes
*NSUN3*	0.21	1.2	(0.76–2)	0.71	0.4	Yes
*NSUN5*	0.02	1	(0.97–1.1)	0.53	0.47	No
*NSUN4*	− 0.04	0.96	(0.72–1.3)	0.07	0.79	Yes
*TET2*	− 0.045	0.96	(0.68–1.4)	0.06	0.8	Yes
*ALYREF*	− 0.0013	1	(0.98–1)	0.02	0.88	Yes
*DNMT3B*	0.031	1	(0.69–1.5)	0.02	0.88	Yes
*DNMT1*	− 0.0011	1	(0.89–1.1)	0	0.99	Yes

Based on the above results, we performed a LASSO technique on the 13 m5C regulatory genes to more precisely identify prognostic markers of PAAD. Through 1,000 LASSO regressions, we observed that LASSO results repeatedly appeared more than 900 times. Only *DNMT3A* exhibited a significant result for expression level and CNV by single factor Cox analysis, and a significant correlation between expression level and clinical grade (*P* < 0.05) (Table 3).

**Table 3:** Results of lasso analysis of m5C regulatory genes.

Duplicates	Genes	CNV express sig	Functions	Survival sig	Stage sig
985	NSUN6	No	Writer	No	No
965	DNMT3A	Yes	Writer	Yes	Yes
939	NSUN2	Yes	Writer	No	No
923	NSUN3	Yes	Writer	No	No
234	DNMT3B	Yes	Writer	No	No
200	TET2	Yes	Eraser	No	No
186	DNMT1	Yes	Writer	No	No
105	NSUN7	No	Writer	No	No
99	NSUN4	Yes	Writer	No	No
88	NSUN5	No	Writer	No	No
86	ALYREF	Yes	Reader	No	No

Given that the *DNMT3A* gene is a writer gene involved in vital m5C regulatory functions and to predict patient risk based on *DNMT3A* gene expression, we analyzed the relationship between *DNMT3A* expression and PAAD patient prognosis. Notably, the results illustrated that low *DNMT3A* expression significantly correlated with poor prognosis (Fig. 5C). These results suggested that *DNMT3A* gene expression could be a clinically significant biomarker for PAAD patients.

### Functional enrichment analysis of *DNMT3A* gene in expression level

Because the *DNMT3A* gene is a methylation writer gene, we next investigated the role of m5C dysfunction in PAAD pathogenesis. We examined enriched gene sets in samples with varying mRNA expression of the *DNMT3A* gene. Gene enrichment analysis by gene set enrichment analysis (GSEA) showed that low *DNMT3A* expression was related to ribosomal processing (Fig. 5D–F). Ribosomes are essential for protein synthesis, and because of the elevated metabolic activity in tumor cells, protein synthesis requirements are much greater than normal cells. These findings also confirmed that the low expression of *DNMT3A* was related to the poor prognosis of patients.

### Validation of *DNMT3A* act as an important pancreatic cancer target tiller invalidation data set

**Figure 6:**
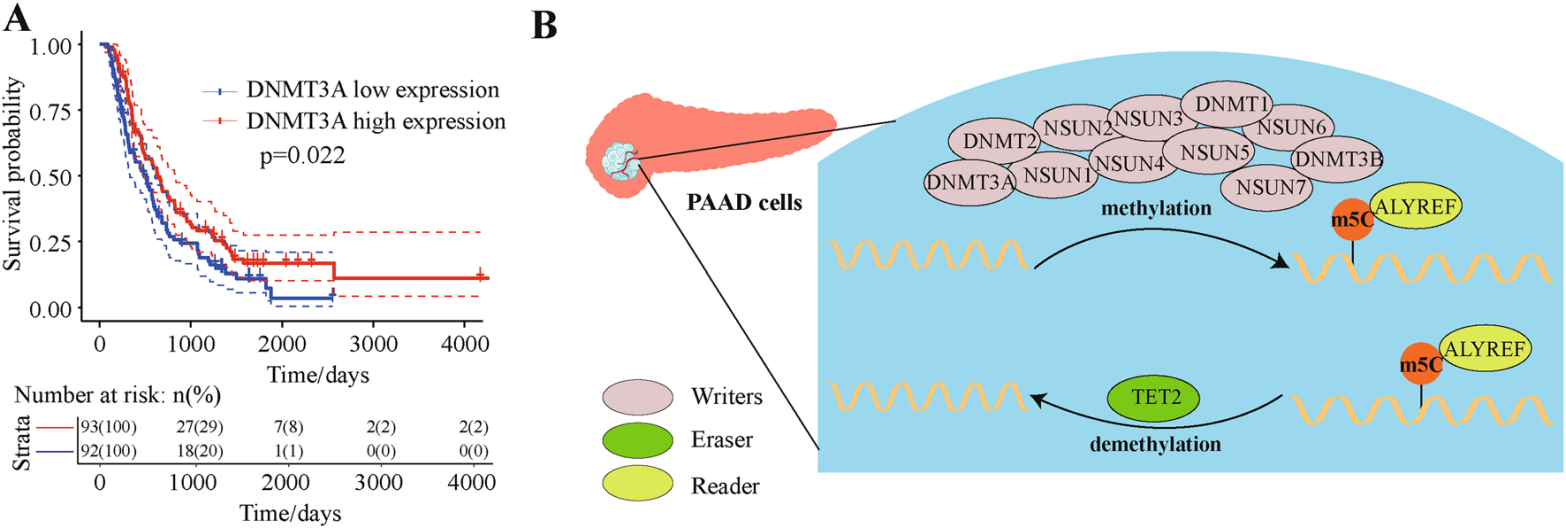
Relationship between *DNMT3A* expression and patient prognosis using validation data set. Analysis of patients from validation dataset ICGC-PACA-CA. (**A**) Relationship between *DNMT3A* expression and patient prognosis in the validation dataset (*P* = 0.022). (**B**) Mechanisms of m5C methylation regulation in PAAD.

Finally, we used a validation data set containing 185 samples to analyze the relationship between *DNMT3A* gene expression and patient survival (Supplementary Table 3). Based on Cox regression analysis, low expression of *DNMT3A* was associated with poor prognosis (Fig. 6A). A schematic diagram showed the related mechanisms of m5C in PAAD cells (Fig. 6B), and these findings provided evidence of the role that m5C epigenetic regulation played in pancreatic cancer.

## Discussion

It is well known that m5C is an important chemical modification to mRNA involved in many pathological processes leading to cancer. The deposition of m5C occurs through a methyltransferase complex involving three homologous factors including methyltransferases known as writers (e.g., NSUN1, NSUN2, NSUN3, NSUN4, NSUN5, NSUN6, NSUN7, DNMT1, DNMT2, DNMT3A, and DNMT3B), demethylases known as erasers (e.g., TET2), and m5C binding proteins known as readers (e.g., ALYREF)^32,33^.

In the present study, we used 686 patients from the TCGA database with sequencing and CNV data to analyze the gene signatures and prognostic values of m5C regulators in PAAD patients. We identified genetic alterations to m5C regulatory genes and patient survival outcomes in pancreatic cancer, and an association between low expression of the writer gene *DNMT3A* and high tumor stage.

These findings provide clues to understanding m5C-mediated epigenetic regulation in pancreatic cancer and perhaps other types of cancer. For example, in patients treated with various chemotherapeutic agents, there was an unexpected and nonspecific amount of DNA methylation activity, as well as a global suppression of DNA repair genes and protein synthesis. Total m5C levels in DNA from patient breast cancer tissue correlates well with tumor malignancy^28^. In one study, cells expressing MYC-TET2 exhibited increased m5C staining, but a concomitant decrease in nuclear m5C staining^30^. In myeloid malignancies, m5C levels could provide valuable diagnostic and prognostic tools to tailor therapies and assess anti-cancer drug responses and toxicity, as *DNMT3A* was recently reported to be a key player in the progression of malignant glioma^19^. In another example, three patients with the highest m5C content in their normal colon appeared to have a germline predisposition to cancer (Lynch syndrome)^29^. Additionally, *DNMT3A* mutations were associated with adverse outcomes among patients with an intermediate-risk cytogenetic profile or *FLT3* mutations, regardless of age, and were independently associated with a poor outcome by Cox proportional-hazards analyses^34^. The high mutation rate of DNMT3A may eventually lead to impaired biological functions, such as cyanosis, because its protein product affects the transmission of m5C signals in cells, which in turn causes tumor-related phenotype changes such as DNA repair activities and oncogenic tyrosine kinases (such as FLT3ITD, BCR-ABL1, JAK2V617F and MPLW515L) activating mutations.

Cancer-related pathways are dysregulated in the development of PAAD. Herein, the expression of *TP53, BRCA1, CDKN2A,* and *ATM* were associated with m5C regulatory gene CNVs and SNVs. Additionally, gene enrichment analysis showed that low *DNMT3A* expression was related to ribosomal processing, an essential part of tumor survival.

In conclusion, we have identified genetic alterations to m5C regulatory genes in pancreatic cancer patients that correlate with patient prognosis and survival, which provides evidence for the role that m5C epigenetic regulation plays in pancreatic cancer.

## Methods

### Data resource and processing

All PAAD clinical, copy number variations (CNVs), simple nucleotide variations (SNVs), and mRNA expression data were retrieved from TCGA-assembler from the TCGA website^35,36^ and downloaded in September 2019. The validation dataset used to validate findings was from the International Cancer Genome Consortium (ICGC) (https://dcc.icgc.org/) using the Pancreatic Cancer Data Set. For transcriptome data, we obtained 177 samples and downloaded data for reading counts. Data were normalized by R package DESeq. For SNV data, we obtained a total of 175 samples, and the downloaded data was level 3 data processed by Mu Tect^37^. For CNV data, there were 183 samples processed by R package RTCGA. Here, the deletion referred to as the Segment Mean was less than 0.2, and the application that was considered as the Segment Mean was greater than 0.2. For clinical data, there were 185 samples after integrating the data and excluding samples with a survival time less than 90 days, leaving a total of 151 pancreatic cancer samples for further analysis.

### The least absolute shrinkage and selection operator (LASSO) analysis

The LASSO method was introduced to identify important predictors of m5C regulatory genes using the R package glmnet as previously described^38,39^. It is a commonly used high-dimensional index regression method that sub-selects the m5C regulatory genes involved in PAAD patient prognosis by applying a penalty proportional to the contraction of the regression coefficient. Based on this method, we constructed m5C regulatory gene signatures with prognostic values, and calculated a risk score for each PAAD patient. All patients were divided into high-risk and low-risk groups with the median risk score as the cut-off score and used Kaplan–Meier curves to estimate the survival of PAAD patients in different groups.

### Gene set enrichment analysis (GSEA)

GSEA is a computational method determining whether an a priori defined set of genes shows statistically significant, concordant differences between two biological phenotypes^40^. GSEA was provided by the JAVA program and downloaded from the website (http://software.broadinstitute.org). We divided all samples into two groups according to the median expression value of the *DNMT3A* gene. Gene sets with a normalized *P* < 0.05 and false discovery rate (FDR) < 0.25 were considered to be significantly enriched.

### Statistical analysis

All statistical data were analyzed using SPSS 23 (IBM, Chicago, USA) and R language. The association between CNV and SNV of m5C regulatory genes and clinicopathological characteristics were analyzed by Chi-square test. The association between three pancreatic cancer Kaplan–Meier curve and we used log-rank test to evaluate the prognosis value of alteration of m5C regulatory gene. All statistical results with a *P*-value less than 0.05 were considered significant.

## Supplementary Information

Supplementary Information.

## Abbreviations

PAAD: Pancreatic adenocarcinoma
m5C: 5-Methylcytosine
TCGA: The Cancer Genome Atlas
CNVs: Copy number variations
SNVs: Simple nucleotide variations
ICGC: International Cancer Genome Consortium
GSEA: Gene set enrichment analysis
FDR: False discovery rate
LASSO: The least absolute shrinkage and selection operator
HR: Hazard ratio
CI: Confidence interval
