# Supplementary material for: Predictive value of m5C regulatory gene expression in pancreatic adenocarcinoma
Source: Sci Rep. 2021 Sep 1;11:17529. doi: 10.1038/s41598-021-96470-w (PMC8410865; doi:10.1038/s41598-021-96470-w)
Supplement: Supplementary file 1 — Supplementary Information. [file 41598_2021_96470_MOESM1_ESM.docx]

**Supplementary Table 1.** **13 PAAD samples with m5C regulatory gene mutations**

| Tags | Days | vital_status | gender | stage_T | stage_N | stage_M | stage | grade |
| --- | --- | --- | --- | --- | --- | --- | --- | --- |
| TCGA-3A-A9IV | 1103 | Dead | Female | T2 | N0 | MX | Stage I | G1 |
| TCGA-3A-A9IN | 2084 | Dead | Female | T2 | NX | MX | Stage I | G2 |
| TCGA-OE-A75W | 267 | Alive | Male | T3 | N0 | M0 | Stage II | G1 |
| TCGA-HZ-8315 | 299 | Alive | Female | T3 | N0 | MX | Stage II | G2 |
| TCGA-2L-AAQI | 103 | Alive | Male | T3 | N1 | MX | Stage II | G3 |
| TCGA-2L-AAQL | 292 | Alive | Male | T3 | N1 | MX | Stage II | G3 |
| TCGA-3A-A9IZ | 308 | Alive | Male | T3 | N1 | MX | Stage II | G2 |
| TCGA-IB-A5SO | 365 | Alive | Male | T3 | N1 | M0 | Stage II | G2 |
| TCGA-HZ-8317 | 378 | Alive | Female | T3 | N1 | MX | Stage II | G1 |
| TCGA-IB-7651 | 603 | Alive | Female | T3 | N1 | M0 | Stage II | G2 |
| TCGA-2J-AABA | 607 | Alive | Male | T3 | N1 | M0 | Stage II | G2 |
| TCGA-HZ-7289 | 661 | Alive | Male | T3 | N1 | MX | Stage II | G1 |
| TCGA-HZ-7918 | 969 | Dead | Male | T3 | N1 | M0 | Stage II | G3 |

**Supplementary Table 2 CNV statistics of m5C-regulated genes in PAAD samples**

| Function | Genes | Diploid | Deletion | Amplification | CNV sum | amplification % | Deletion% | Percentage |
| --- | --- | --- | --- | --- | --- | --- | --- | --- |
| writer | *NSUN1* | - | - | - | - | - | - | - |
|  | *NSUN2* | 159 | 5 | 20 | 25 | 80.00% | 20.00% | 13.59% |
|  | *NSUN3* | 167 | 8 | 9 | 17 | 52.94% | 47.06% | 9.24% |
|  | *NSUN4* | 156 | 25 | 3 | 28 | 10.71% | 89.29% | 15.22% |
|  | *NSUN5* | 161 | 2 | 20 | 22 | 90.91% | 9.09% | 12.02% |
|  | *NSUN6* | 161 | 19 | 4 | 23 | 17.39% | 82.61% | 12.50% |
|  | *NSUN7* | 169 | 5 | 10 | 15 | 66.67% | 33.33% | 8.15% |
|  | *DNMT1* | 164 | 13 | 7 | 20 | 35.00% | 65.00% | 10.87% |
|  | *DNMT2* | - | - | - | - | - | - | - |
|  | *DNMT3A* | 165 | 15 | 4 | 19 | 21.05% | 78.95% | 10.33% |
|  | *DNMT3B* | 156 | 2 | 26 | 28 | 92.86% | 7.14% | 15.22% |
| eraser | *TET2* | 175 | 7 | 5 | 12 | 41.67% | 58.33% | 6.42% |
| reader | *ALYREF* | 150 | 23 | 11 | 34 | 32.35% | 67.65% | 18.48% |

**Supplementary Table 3. The relationship between DNMT3A gene expression and patient survival in 185 samples**

| **Tags** | **Days** | **vital_status** | **DonorID** | **Gender** | **Age** | **TNM** |
| --- | --- | --- | --- | --- | --- | --- |
| SP197558 | 65 | Alive | DO224758 | male | 58 | IIB |
| SP196616 | 90 | Alive | DO35132 | male | 69 | IIA |
| SP125808 | 102 | Alive | DO35184 | male | 75 | IB or IIB |
| SP113873 | 107 | Alive | DO51483 | female | 67 | IIB |
| SP197716 | 113 | Alive | DO227570 | male |  |  |
| SP197398 | 129 | Alive | DO51518 | female | 66 | IB |
| SP197688 | 132 | Alive | DO227544 | male |  |  |
| SP117722 | 141 | Alive | DO51540 | female | 69 | IIA |
| SP192353 | 142 | Alive | DO227596 | male |  |  |
| SP133632 | 144 | Alive | DO51464 | male | 72 | IIB |
| SP117643 | 146 | Alive | DO35350 | male | 76 | IB |
| SP192385 | 158 | Alive | DO227736 | male |  |  |
| SP196620 | 160 | Alive | DO35406 | male | 54 | III |
| SP197538 | 164 | Alive | DO227604 | female |  |  |
| SP133884 | 165 | Alive | DO224750 | female | 52 | IIB |
| SP197581 | 170 | Alive | DO230464 | female |  |  |
| SP192351 | 177 | Alive | DO227581 | female |  |  |
| SP125761 | 184 | Alive | DO51512 | male | 42 | IIA |
| SP197535 | 190 | Alive | DO51545 | female | 75 | IB |
| SP197681 | 192 | Alive | DO227531 | male |  |  |
| SP125713 | 195 | Alive | DO221541 | male | 75 | IA |
| SP197382 | 197 | Alive | DO51496 | female | 70 | IIB |
| SP133937 | 200 | Dead | DO224770 | female | 74 | IA |
| SP125785 | 201 | Alive | DO51522 | female | 50 | IIA |
| SP196657 | 201 | Alive | DO35242 | male | 51 | T3N1Mx |
| SP197568 | 201 | Alive | DO224776 | male | 70 | IB |
| SP77904 | 209 | Alive | DO35160 | female | 48 | TxNxMx |
| SP197700 | 216 | Alive | DO227558 | male |  |  |
| SP197712 | 217 | Alive | DO227564 | male |  |  |
| SP125782 | 224 | Alive | DO35085 | male | 59 | IIA |
| SP113743 | 228 | Alive | DO51480 | female | 41 | IIB |
| SP133951 | 228 | Alive | DO224779 | female | 69 | IB |
| SP125766 | 229 | Dead | DO51549 | female | 76 | IIA |
| SP125694 | 232 | Alive | DO51490 | female | 78 | IIA |
| SP78286 | 233 | Alive | DO35100 | male | 78 | T3N0M0 |
| SP192364 | 234 | Alive | DO224724 | female | 63 | IB |
| SP133812 | 239 | Alive | DO224712 | male | 78 | IIB |
| SP133914 | 244 | Dead | DO224764 | male | 58 | IIA |
| SP125729 | 247 | Alive | DO35138 | female | 69 | IIB |
| SP133895 | 257 | Dead | DO224752 | male | 67 | IB or IIB |
| SP125778 | 260 | Dead | DO221545 | male | 63 | IB |
| SP125753 | 263 | Alive | DO35230 | female | 52 | IIB |
| SP133858 | 271 | Alive | DO224734 | male | 62 | IB |
| SP125768 | 276 | Alive | DO51529 | male | 87 | IIB |
| SP113900 | 278 | Alive | DO51534 | female | 79 | IIB |
| SP117463 | 280 | Alive | DO35126 | female | 55 | IIB |
| SP117351 | 282 | Alive | DO51492 | female | 57 | IIA |
| SP125706 | 287 | Alive | DO51510 | male | 43 | IIB |
| SP125807 | 290 | Alive | DO221540 | male | 56 | IIB |
| SP197505 | 294 | Alive | DO51498 | female | 40 | III |
| SP117523 | 294 | Alive | DO49454 | female | 74 | II |
| SP125731 | 294 | Alive | DO51501 | male | 53 | IIB |
| SP125714 | 310 | Alive | DO51502 | male | 47 | Unknown |
| SP192365 | 311 | Alive | DO227648 | female |  |  |
| SP117878 | 311 | Alive | DO35144 | male | 69 | IB or IIB |
| SP125693 | 311 | Alive | DO51478 | male | 69 | IB or IIB |
| SP125712 | 313 | Alive | DO51497 | female | 70 | IIA |
| SP117340 | 314 | Alive | DO51469 | female | 80 | IB |
| SP125757 | 315 | Alive | DO35454 | female | 74 | IIA |
| SP125730 | 318 | Alive | DO51523 | male | 48 | IB |
| SP133925 | 324 | Alive | DO224767 | male | 48 | IB |
| SP125737 | 325 | Alive | DO51541 | male | 81 | IIB |
| SP125718 | 330 | Alive | DO35152 | male | 55 | IB |
| SP197357 | 341 | Alive | DO49424 | male | 84 | IA |
| SP125763 | 349 | Alive | DO51527 | female | 86 | IA |
| SP117037 | 351 | Alive | DO51505 | female | 64 | IB or IIB |
| SP125758 | 351 | Alive | DO51466 | male | 69 | IIA |
| SP117908 | 361 | Alive | DO35128 | female | 83 | IB |
| SP125711 | 361 | Alive | DO51538 | female | 76 | IIB |
| SP117216 | 362 | Alive | DO35258 | male | 82 | IIA |
| SP197519 | 369 | Alive | DO51542 | female | 54 | IIB |
| SP117323 | 371 | Alive | DO35082 | male | 68 | IIB |
| SP125769 | 375 | Dead | DO221542 | male | 60 | IIA |
| SP113884 | 379 | Alive | DO51482 | male | 73 | IB |
| SP197405 | 383 | Alive | DO49481 | male | 64 | II |
| SP108706 | 385 | Alive | DO49421 | female | 74 | IIB |
| SP133956 | 389 | Alive | DO224782 | female | 59 | III |
| SP197521 | 400 | Dead | DO51513 | female | 85 | IB |
| SP192363 | 437 | Alive | DO224714 | male | 73 | IB |
| SP125717 | 444 | Alive | DO51470 | female | 51 | IIB |
| SP125762 | 446 | Alive | DO51536 | female | 82 | IIB |
| SP133802 | 455 | Alive | DO224705 | female | 59 | IIB |
| SP108735 | 456 | Alive | DO49433 | male | 48 | IIB |
| SP197552 | 456 | Alive | DO224745 | male | 55 | IIB |
| SP125776 | 463 | Alive | DO35198 | female | 70 | IIA |
| SP125804 | 467 | Dead | DO221544 | male | 42 | IIA |
| SP125696 | 471 | Alive | DO51467 | male | 67 | III |
| SP133784 | 472 | Alive | DO224698 | male | 72 | IIA |
| SP125784 | 480 | Alive | DO221546 | male | 61 | IIB |
| SP125752 | 482 | Dead | DO51500 | female | 69 | Unknown |
| SP192388 | 482 | Alive | DO227742 | male |  |  |
| SP125783 | 494 | Alive | DO51504 | female | 67 | IB |
| SP197509 | 503 | Alive | DO51537 | male | 73 | IIA |
| SP113837 | 512 | Alive | DO51493 | female | 52 | IIB |
| SP113796 | 515 | Alive | DO51509 | male | 66 | IIB |
| SP133832 | 522 | Alive | DO224719 | male | 67 | III |
| SP192377 | 531 | Alive | DO227695 | female |  |  |
| SP197529 | 539 | Dead | DO51491 | male | 83 | IB |
| SP197366 | 547 | Alive | DO224642 | female | 57 | IIB |
| SP197387 | 573 | Alive | DO49469 | male | 78 | IB |
| SP197476 | 574 | Alive | DO230463 | male |  |  |
| SP196608 | 575 | Alive | DO35424 | male | 74 | IIB |
| SP197392 | 578 | Alive | DO49472 | male | 66 | I-III |
| SP125795 | 579 | Alive | DO51519 | female | 70 | IIB |
| SP125760 | 587 | Alive | DO51481 | female | 59 | IIA |
| SP125685 | 590 | Alive | DO51472 | female | 65 | IB |
| SP197515 | 592 | Alive | DO51511 | female | 67 | IIA |
| SP116985 | 608 | Alive | DO49439 | female | 79 | IIB |
| SP125698 | 625 | Alive | DO51530 | female | 43 | III |
| SP125799 | 627 | Alive | DO35226 | female | 50 | IA |
| SP197401 | 628 | Alive | DO49475 | male | 74 | IB |
| SP113865 | 631 | Alive | DO51494 | male | 59 | IIB |
| SP133764 | 633 | Alive | DO224688 | male | 61 |  |
| SP108828 | 653 | Dead | DO49478 | male | 81 | IB |
| SP117911 | 663 | Alive | DO49436 | female | 69 | IIB |
| SP192371 | 673 | Alive | DO227671 | female |  |  |
| SP125699 | 673 | Alive | DO35290 | male | 65 | IIB |
| SP125764 | 681 | Alive | DO49419 | male | 50 | I-III |
| SP197585 | 681 | Alive | DO227704 | male |  |  |
| SP133866 | 684 | Alive | DO224740 | male | 63 | IB |
| SP125793 | 690 | Alive | DO51525 | male | 38 | IB |
| SP117656 | 729 | Alive | DO51495 | male | 66 | IIB |
| SP117290 | 730 | Alive | DO49463 | female | 78 | IB |
| SP197362 | 742 | Alive | DO224633 | male | 72 | IIA |
| SP192373 | 745 | Alive | DO227684 | female |  |  |
| SP117440 | 746 | Alive | DO35305 | male | 70 | IB |
| SP113875 | 764 | Alive | DO51535 | male | 84 | IIB |
| SP192350 | 767 | Alive | DO49460 | female | 64 | IB |
| SP133962 | 816 | Alive | DO224784 | female | 72 | I - III |
| SP117002 | 821 | Alive | DO49430 | male | 81 | IIA |
| SP192375 | 823 | Alive | DO227687 | male |  |  |
| SP191680 | 826 | Alive | DO224575 | male | 72 | IIB |
| SP125738 | 837 | Alive | DO51503 | female | 46 | IIB |
| SP196645 | 886 | Alive | DO35210 | male | 75 |  |
| SP133678 | 889 | Dead | DO35365 | male | 61 |  |
| SP197376 | 904 | Dead | DO49457 | female | 53 | IB |
| SP197372 | 918 | Alive | DO49451 | male | 66 | IB |
| SP125739 | 951 | Alive | DO49448 | male | 68 | IB |
| SP133708 | 958 | Alive | DO224648 | male | 69 | IIB |
| SP117980 | 995 | Alive | DO49442 | male | 57 | IIB |
| SP196669 | 1013 | Alive | DO35148 | female | 77 | IB or IIB |
| SP125750 | 1068 | Alive | DO51506 | female | 62 | II |
| SP125695 | 1072 | Alive | DO35496 | female | 63 | IB or IIB |
| SP197544 | 1074 | Alive | DO227633 | male |  |  |
| SP113727 | 1083 | Alive | DO51468 | male | 69 | III |
| SP125787 | 1091 | Alive | DO35104 | male | 88 | Unknown |
| SP117261 | 1164 | Dead | DO49420 | male | 64 | IIB |
| SP117949 | 1191 | Alive | DO51514 | male | 61 | IB |
| SP133731 | 1208 | Alive | DO224656 | female | 50 | IB |
| SP125716 | 1226 | Alive | DO51465 | female | 74 | IB |
| SP125772 | 1234 | Alive | DO35222 | male | 53 | IB |
| SP125725 | 1251 | Dead | DO51524 | male | 79 | IIA |
| SP118083 | 1253 | Dead | DO51489 | female | 60 | IIB |
| SP125687 | 1262 | Alive | DO35140 | female | 54 | IA |
| SP125746 | 1264 | Dead | DO35083 | female | 68 | IIB |
| SP125741 | 1273 | Alive | DO51531 | male | 48 | IIB |
| SP125732 | 1331 | Dead | DO35442 | female | 53 | IIA |
| SP117568 | 1343 | Dead | DO35236 | female | 58 | IB |
| SP125724 | 1347 | Dead | DO35136 | female | 61 | IB |
| SP117076 | 1359 | Alive | DO35330 | male | 63 | IIA |
| SP113829 | 1380 | Alive | DO51526 | female | 82 | IIB |
| SP133674 | 1389 | Alive | DO51517 | male | 52 | IIB |
| SP125780 | 1431 | Alive | DO51543 | female | 76 | IB or IIB |
| SP118048 | 1448 | Alive | DO49418 | male | 58 | IA |
| SP125710 | 1460 | Alive | DO51484 | female | 63 | IA |
| SP117690 | 1483 | Dead | DO49427 | female | 69 | IIA |
| SP196624 | 1499 | Alive | DO35098 | male | 63 | IB or IIB |
| SP125756 | 1576 | Alive | DO51476 | female | 45 | IIA |
| SP125781 | 1623 | Dead | DO35228 | male | 68 | IA |
| SP125770 | 1633 | Dead | DO51474 | male | 63 | IB |
| SP125771 | 1646 | Dead | DO35116 | female | 76 | IB |
| SP125719 | 1691 | Dead | DO51479 | male | 70 | Unknown |
| SP125703 | 1733 | Dead | DO221539 | male | 49 | IIB |
| SP125802 | 1761 | Dead | DO51532 | female | 59 | IB |
| SP125755 | 1799 | Dead | DO51515 | male | 73 | IB or IIB |
| SP125797 | 1824 | Alive | DO51475 | female | 58 | IA |
| SP117476 | 1879 | Alive | DO51485 | female | 74 | IIB |
| SP125722 | 2045 | Dead | DO221543 | male | 54 | IIB |
| SP125742 | 2174 | Dead | DO35360 | male | 65 | IB |
| SP125809 | 2177 | Dead | DO51548 | female | 55 | IIB |
| SP125686 | 2325 | Dead | DO51528 | male | 62 | IIB |
| SP125723 | 2555 | Dead | DO51487 | female | 69 | IIB |
| SP117760 | 2571 | Alive | DO51507 | male | 73 | IIB |
| SP125811 | 4173 | Dead | DO51533 | male | 67 | IB |
| SP117215 | 4289 | Alive | DO51473 | female | 76 | IIB |

**Supplementary Table 4** **GSEA enrichment analysis results of *DNMT3A***

| NAME | ES | NES | NOM p-val | FDR p-val | FWER p-val |
| --- | --- | --- | --- | --- | --- |
| SMALL RIBOSOMAL SUBUNIT | -0.7593395 | -1.8760052 | 0.00195695 | 1 | 0.737 |
| SRP-DEPENDENT COTRANSLATIONAL PROTEIN TARGETING TO MEMBRANE | -0.8327598 | -1.8622003 | 0.00195313 | 0.9004415 | 0.769 |
| FORMATION OF THE TERNARY COMPLEX, AND SUBSEQUENTLY, THE 43S COMPLEX | -0.832807 | -1.8469983 | 0.00395257 | 0.7150872 | 0.806 |
| L13A-MEDIATED TRANSLATIONAL SILENCING OF CERULOPLASMIN EXPRESSION | -0.8162577 | -1.8202875 | 0.0077821 | 0.7323666 | 0.884 |
| GTP HYDROLYSIS AND JOINING OF THE 60S RIBOSOMAL SUBUNIT%REACTOME DATABASE ID RELEASE 65 | -0.803862 | -1.8024815 | 0.01171875 | 0.7248055 | 0.914 |
| STRUCTURAL CONSTITUENT OF RIBOSOME | -0.7814668 | -1.7963976 | 0.00200803 | 0.6484093 | 0.922 |
| ACTIVATION OF THE MRNA UPON BINDING OF THE CAP-BINDING COMPLEX AND EIFS, AND SUBSEQUENT BINDING TO 43S%REACTOME DATABASE ID RELEASE | -0.7460665 | -1.7951391 | 0.01380671 | 0.56488925 | 0.923 |
| FORMATION OF A POOL OF FREE 40S SUBUNITS%REACTOME DATABASE ID RELEASE | -0.8668051 | -1.7873499 | 0.00396825 | 0.5391322 | 0.934 |
| TRANSLATION INITIATION COMPLEX FORMATION | -0.7398917 | -1.7770138 | 0.01771654 | 0.53448606 | 0.942 |
| PROTEIN TARGETING TO ER | -0.8460634 | -1.772373 | 0.00592885 | 0.5037274 | 0.948 |
| PROTEIN LOCALIZATION TO ENDOPLASMIC RETICULUM | -0.7542943 | -1.7689879 | 0.00986193 | 0.4739752 | 0.951 |
| COTRANSLATIONAL PROTEIN TARGETING TO MEMBRANE | -0.8581105 | -1.7685306 | 0.00394477 | 0.43644 | 0.951 |
| CAP-DEPENDENT TRANSLATION INITIATION%REACTOME | -0.7623071 | -1.7664235 | 0.02697495 | 0.41236743 | 0.954 |
| EUKARYOTIC TRANSLATION INITIATION | -0.7623071 | -1.7664235 | 0.02697495 | 0.3829126 | 0.954 |
| CYTOSOLIC SMALL RIBOSOMAL SUBUNIT | -0.8043378 | -1.7656513 | 0.00970874 | 0.3607624 | 0.954 |
| SELENOAMINO ACID METABOLISM | -0.7707048 | -1.7618513 | 0.01584159 | 0.3524669 | 0.957 |
| CYTOSOLIC RIBOSOME | -0.7856267 | -1.7608757 | 0.00980392 | 0.3350597 | 0.96 |
| RIBOSOMAL SCANNING AND START CODON RECOGNITION | -0.7193037 | -1.7555941 | 0.02140078 | 0.33337432 | 0.963 |
| ESTABLISHMENT OF PROTEIN LOCALIZATION TO ENDOPLASMIC RETICULUM | -0.8053879 | -1.7521458 | 0.00982318 | 0.32732996 | 0.967 |
| EUKARYOTIC TRANSLATION ELONGATION | -0.8876641 | -1.7513913 | 0.00393701 | 0.31389323 | 0.967 |
